# Effort Displayed During Appetitive Phase of Feeding Behavior Requires Infralimbic Cortex Activity and Histamine H1 Receptor Signaling

**DOI:** 10.3389/fnins.2019.00577

**Published:** 2019-06-28

**Authors:** María E. Riveros, María Ines Forray, Fernando Torrealba, José L. Valdés

**Affiliations:** ^1^Departamento de Ciencias Fisiológicas, Facultad de Ciencias Biológicas, Pontificia Universidad Católica de Chile, Santiago, Chile; ^2^Centro de Fisiología Integrativa, Facultad de Medicina, Clínica Alemana Universidad del Desarrollo, Santiago, Chile; ^3^Center of Applied Ecology and Sustainability, Santiago, Chile; ^4^Departamento de Química, Pontificia Universidad Católica de Chile, Santiago, Chile; ^5^Departamento de Neurociencias, Facultad de Medicina, Universidad de Chile, Santiago, Chile; ^6^Biomedical Neuroscience Institute, Facultad de Medicina, Universidad de Chile, Santiago, Chile

**Keywords:** histmaine, prefrontal cortex, infralimbic cortex, microdialysis, effort, motivation

## Abstract

The chances to succeed in goal-directed behaviors, such as food or water-seeking, improve when the subject is in an increased arousal state. The appetitive phase of these motivated behaviors is characterized by high levels of behavioral and vegetative excitation. The key decision of engaging in those particular behaviors depends primarily on prefrontal cortical areas, such as the ventromedial prefrontal cortex. We propose that the infralimbic cortex (ILC) located in the medial prefrontal cortex induces an increase in arousal during the appetitive phase of motivated behavior, and that this increase in arousal is, in turn, mediated by the activation of the brain histaminergic system, resulting in higher motivation for getting food rewards. To test this hypothesis, we conduct a progressive ratio operant conditioning to test the degree of motivation for food, while simultaneously manipulating the histaminergic system through pharmacologic interventions. We found that the behavioral responses to obtain food in hungry rats were disrupted when the ILC was inhibited through muscimol infusion, blocking brain H1 histamine receptors by intracerebroventricular infusion of pyrilamine or by satiety. In contrast, the consummatory behavior was not affected by ILC inhibition. The extracellular histamine levels in the ILC were increased in direct correlation with the degree of motivation measured in the progressive ratio test. ILC inhibition also prevented this increase in histamine levels. The rise in extracellular histamine levels during the progressive ratio test was similar (*ca.* 200%) during the active or the resting period of the day. However, different basal levels are observed for these two periods. Our findings suggest that increased histamine levels during this behavior are not simply explained by the awaked state, but instead, there is a motivation-related release of histamine, suggestive of a specific form of brain activation. Serotonin (another critical component of the ascending arousal system) was also tested. Interestingly, changes in levels of this neuromodulator were not detected during the progressive ratio test. In conclusion, our results suggest that ILC activation and subsequent increase in brain histamine release are both necessary for the normal performance of a motivated behavior such as feeding.

## Introduction

In order to maximize advantageous actions, higher-order organisms need to display a continuous trade-off analysis of potential benefits and threats coming from the environment, along with development of the ability to balance these benefits with costs of executing actions to obtain positive reinforcers or avoid and prevent potential damage (Batten and Shoemaker, 1961). The decision to engage in an action directed to a specific goal requires a “motivated state.” Motivation, thus, increases the probability of performing a particular goal-directed behavior.

Two key aspects of motivation have been proposed by Hebb (1955). One is direction, related to the specificity of actions that will be selected, and primarily driven by an internal state of the individual; the second is intensity, related to the vigor and persistence of the actions performed during a motivated state. The motivational intensity of behavior has been consistently linked to an increase in the thalamocortical activity (behavioral arousal) and the autonomic activity (vegetative arousal), implicating a change in the global state of brain activity (Kinomura et al., 1996; Hirata and Castro-Alamancos, 2010; Meeusen et al., 2013; Liu et al., 2015).

The global state of the mammalian brain changes to accommodate its functioning mode to the individual current needs, alternating among sleep and awake states. During the awake state, the level of arousal variates and is related to the performance in a U-shaped curve (Yerkes and Dodson, 1908; Mair et al., 2011). Therefore, increasing arousal increases performance; for example, it has been reported that a moderate increase in arousal induced by exercise is associated with an increase in cognitive performance (Murray and Russoniello, 2012) However, this relation reaches an optimal arousal state for performance, but beyond this optimal state, further increases in arousal will worsen performance. Thus, adjusting arousal state to performance demands is part of behavioral regulation needed during a motivated behavior. Levels of arousal are adjusted by neuromodulator systems, commonly known altogether as the ascending arousal system (AAS) (Jones, 2003). Because the nuclei that form part of this system have extensive connections to the entire brain, the AAS is capable of inducing changes in general levels of activity in the brain. In mice, the level of wakefulness is strongly related to the electrical activity of the histaminergic cells of the tuberomamillary nucleus (TMN) (Takahashi et al., 2006). Dopamine has been consistently shown to be involved in behavioral changes induced by motivation, such as the increase in the vigor of response (Sokolowski and Salamone, 1998; Aberman and Salamone, 1999; Salamone et al., 2001; Correa et al., 2002; Mingote et al., 2005; Beierholm et al., 2013), attribution of salience, and learning of reward value (Berridge and Robinson, 1998). However, the role of histamine in arousal regulation during motivated behavior has been less explored. We have reported previously that TMN histaminergic neurons are the first component of the AAS to get activated during the appetitive phase of a food-related motivated behavior (Valdés et al., 2005) paralleled with an increase in locomotion (behavioral arousal), body core temperatures (vegetative arousal), and the activation of the infralimbic cortex (ILC) (Valdés et al., 2006). The ILC is reciprocally connected to the TMN, through projections that connect to the histaminergic neurons of the TMN (Wouterlood et al., 1987; Vertes, 2004) constituting the principal excitatory input to the TMN (Ericson et al., 1991) and, in turn, receiving back projection fibers from the histaminergic neurons (Chauveau et al., 2014). Also, histamine is released in the hypothalamus and ILC during the appetitive phase of feeding behavior (Valdés et al., 2010; Riveros et al., 2014). ILC pharmacological activation induces histamine release at this same cortical region in agreement with the anatomical reciprocal connection (Riveros et al., 2015). These findings indicate an essential role of both TMN and ILC in the regulation of behavioral and vegetative arousal during food-related appetitive behavior.

To better quantify the relationship between motivation and brain histaminergic activity, we have evaluated the effort that rats make in order to obtain food rewards (Salamone et al., 2009, 2012; Pardo et al., 2012.

Dopamine has shown to be an essential mediator between motivation and effort (Salamone et al., 2012), as it has been demonstrated by the reduction in willingness to work after treatment with dopamine receptor antagonists such as haloperidol (Pardo et al., 2012). This effect of haloperidol is reversed by caffeine, an adenosine 2 receptor antagonist (Salamone et al., 2009; Pardo et al., 2012). Interestingly, caffeine also activates histaminergic neurons in the TMN and increases histamine release (John et al., 2014), while an agonist of the adenosine 2 receptor reduces histamine release, inducing sleep (Hong et al., 2005), suggesting that during goal-directed behavior and downstream of dopaminergic pathways, a histaminergic mechanism could mediate the increase in motivation intensity.

We hypothesized that histamine neurotransmission driven by ILC enables energy mobilization to increase the willingness to make an effort, possibly by reducing effort perception. This interpretation is supported by studies in humans showing that lesions in the ventromedial prefrontal cortex (vmPFC) increase the effort perception (Pardini et al., 2010). In keeping with these findings, injury of the medial prefrontal cortex in rats shifts the animal decisions toward less effortful and less rewarding choices (Walton et al., 2002).

To test whether this hypothesis underlies our and others prior observations, we tested whether activation of the histaminergic system during the appetitive phase was related to the willingness to exert effort in exchange for food rewards. We assessed the rate of operational response in the progressive ratio task of motivated animals, when H1 histamine receptors were blocked, when the ILC was pharmacologically inactivated (Riveros et al., 2014), or when the animals were not motivated (satiated), while levels of histamine release and serotonin were measured simultaneously.

## Materials and Methods

### Animals

We used male adult Sprague-Dawley male rats, weighing 270–350 g. They were individually housed in a controlled environment at 23°C, with 12 h/12 h lights on/off schedule (lights off at 1900 h), and had permanent access to water and food, except when it was indicated. All experiments were carried out at Pontificia Universidad Católica de Chile Animal Care Facility in concordance to the NIH (United States) *Guide for the Care and Use of Laboratory Animals* (NIH Publications No. 80-23, revised 1996). Our local institutional Bio-Safety and Ethical Committee approved these experimental protocols, which minimized the number of rats used and their suffering.

### Operant Conditioning to Evaluate the Effort

We used a modified version of the progressive ratio test, where the animals learn to press a lever to get rewards. In our version of the test (in the first step, the motivation test), animals do not get any reward after pressing the lever, preventing the consummatory phase of the behavior and sustaining the appetitive phase. The number of lever presses until the animal gives up was used as an indicator of motivation. The rat operant conditioning apparatus (Med-Associates, Inc., Alpharetta, GA, United States model ENV-008CT) consists of a chamber with a stainless-steel grid floor, ventilated and sound attenuated. Each chamber had a pellet dispenser, a house light that remained turned on during the experiments, a pellet receptacle, and two retractable levers with a light signal over them; one light over the lever was turned on for 10 s when pellets were available. Rats were trained 2 h/day for 3 days to press a lever to obtain a chocolate flavored pellet (45 mg/pellet, product # FO299; Bio-Serv, Flemington, NJ, United States) at a ratio of one pellet per lever press. On the 4th day of training, the ratio increased to 3:1, to 5:1 on the 5th day, to 10:1 on the 6th day, and to 15:1 on the 7th day. On the 10th day (test day), the rats were divided into three groups: one group was fasted for 24 h (*n* = 13) and injected with 1 μl of saline bilaterally in the ILC. A second group was fed *ad libitum* (*n* = 8) and injected with 1 μl of saline bilaterally in the ILC. The third group (*n* = 10) fasted for 24 h, and pretreated with 0.5-μl microinjections of muscimol (Sigma-Aldrich, Co., St. Louis, MO, United States) 100 ng/μl bilaterally in the ILC, 10 min before the test. For a different experiment, three groups (27 animals) were used, two groups received pyrilamine (Cat N: Sigma-Aldrich, Co., St. Louis, MO, United States), a histamine H1 receptor inverse agonist, that was delivered intraperitoneally in doses of 20 mg per kilogram of body weight for one group (*n* = 9) and 30 mg per kilogram of body weight for the second group (*n* = 9), while a third group (*n* = 9) was intraperitoneally injected with saline as control vehicle. All injections were performed 30 min before the test. For this experiment, the two available levers were extended during training and test phases. In this setting, pressing only one of them resulted in the delivery of one pellet, so pressing the non-reward associated lever was registered as an error during the test session. Position of the correct lever remained stable and was counterbalanced between animals, so half of them had left and half had right as the correct lever.

Each rat was tested only once to avoid extinction of the operant behavior. During the 30-min test, the light above the lever was on, signaling food availability, but no pellets were delivered. The number of lever presses indicated how much the rats were willing to work or motivated for obtaining rewards. As previously said, we did not use a conventional progressive ratio protocol to avoid that the animals reach the consummatory phase before proceeding to the next step, where we measured the intake of chocolate pellets. After the test, the rats remained in the apparatus, lights were off and the lever was retracted and then immediately lights were on again and the lever extended, then one pellet was automatically delivered, and for a 30-min period, one chocolate pellet was now delivered per lever press. The number of lever presses in this stage was considered as pellets ingested. We confirmed that all pellets delivered were eaten, and if pellets were left in the cage, they were subtracted from the number of lever presses to calculate ingested pellets and the automatically delivered pellet was added. All the above explained procedures were performed during the light phase of the circadian cycle.

To determine the influence of circadian phase (subjective day or night) on the behavioral performance and histamine release, this procedure was repeated in other groups of rats (*n* = 16), but under an inverted circadian cycle schedule during 2 weeks of housing before testing.

### Surgery

Rats were anesthetized with 100 mg/kg i.p. of ketamine (Imalgene^TM^, Rhodia Merieux, Santiago) plus 20 mg/kg of xylazine (Rompun^TM^, Bayer, Santiago). The animals were placed in a stereotaxic frame (S1600 model, Stoelting, Co., Wood Dale, IL, United States). The head was fixed and positioned, so that bregma and lambda were in the same horizontal plane. Guide cannulae for microdialysis or microinjection were implanted bilaterally in the ILC of the vmPFC at the following coordinates (Swanson, 1998): 3.0 mm anterior to bregma, 0.5 mm lateral, and 3.6 mm below the duramater; and in both lateral ventricles at 3.4 mm posterior to bregma, ± 1.5 mm lateral, and 4.4 mm below the dura mater, for H1 receptor inverse agonist injection. The microdialysis guide cannulae (MD2251, BASi, Lafayette, IN, United States) consist of a guide head and an occluder, which were fixed to the skull with dental acrylic (Marche^TM^, Santiago, Chile), stainless-steel jewellery screws, and plastic support. One of the cannula guides was implanted in one IL at the above coordinates, and the other was implanted in the contralateral IL using a 24° angle in the anteroposterior axis. After guide cannula implantation, the animals were individually housed for 7 days before brain microdialysis started. Enrofloxacin 5% (19 mg/kg i.p., Bayer Santiago) and Ketophen (0.2 mg/kg i.p., Rhodia Merieux) were administrated at the end of surgery as antibiotic and anti-inflammatory measures, respectively.

### Histamine Monitoring in the vmPFC of Freely Moving Rats

We performed microdialysis in awake and freely moving animals while they were tested in the operant chamber. We used either a concentric microdialysis probe of 2 mm length, 0.5 mm outer diameter, cutoff 30,000 kDa (BASi, Lafayette, IN, United States), or a combination probe (BASi, MD2262) that allowed muscimol infusion in addition to performing microdialysis. The probe was lowered 2 mm below the cannulae guide tip into the ILC. The probe was continuously perfused at a rate of 2 μl/min with artificial cerebrospinal fluid (ACSF) using a microperfusion pump (KDS100L model from KD Scientific, Inc., Holliston, MA, United States). The composition of the ACSF solution was 147 mM NaCl, 1.2 mM CaCl_2_, 4 mM KCl, 2 mM Na_2_HPO_4_, and 0.2 mM NaH_2_ PO_4_ (pH 7.4), freshly prepared with sterile distilled water. After a stabilization period of 120 min, three basal samples of 10 min each were collected in separate tubes containing 2.0 μl of 0.2 N perchloric acid, after which samples were collected every 10 min during all experimental protocols.

### Derivatization for Histamine and Serotonin

Ten microliters of borate buffer 0.5 M and 5 μl of the fresh solution [4 mg ophtalaldehyde (OPA) and 2 μl of β-mercaptoethanol in 1 ml of methanol] were added to 20 μl of the microdialysis sample for derivatization. The mixture was agitated for 30 s and injected after 1 min in the HPLC column (LiChroCART 250-4 Purospher STAR RP-18 endcapped, particle size 5 μm; Merck KGaA, Darmstadt, Germany). The mobile phase contained phosphate buffer (0.1 mol/L KH_2_PO_4_) and an acetonitrile gradient from 25 to 50% and was set at a flux of 1 ml per minute. Acetonitrile and phosphate buffer were automatically mixed by the pump (Elite labChrom L-2130; Hitachi, Chiyoda, Tokyo, Japan). The resultant fluorescent derivatives were analyzed using a fluorometric detector (Elite labChrom L-2480; Hitachi). We used excitation/emission wavelengths of 330/450 nm, respectively. Histamine and serotonin (from Sigma-Aldrich, Co.) standards dissolved in ACSF in a concentration range from 0.06 to 0.25 pmol/20 μl were used to construct calibration curves that produced a linear correlation between neurotransmitter concentration and peak area.

### Statistical Analysis

Extracellular levels of histamine and serotonin were expressed as the mean ± SEM of the percentage of basal levels. The time courses of the effect of IL inactivation (muscimol) on histamine and serotonin extracellular levels were statistically analyzed by a two-way repeated-measures analysis of variance (ANOVA) followed by Bonferroni *post hoc* tests, with drug treatment and time as factors. The number of lever presses and pellets eaten was analyzed using a one-way ANOVA, followed by a Tukey post-test. The number of lever presses was analyzed by one-tailed *t*-test with Welch’s correction. The absolute values of histamine and serotonin extracellular levels were analyzed by a two-tailed *t*-test with Welch’s correction. The statistical analysis was performed using GraphPad Prism software.

## Results

### Decreased ILC-TMN Activity Reduces the Effort to Get a Reinforcer

In order to have a direct assessment of motivation, we evaluated the effort displayed to obtain a reinforcer (that is, during appetitive behavior) in rats trained on an operant chamber. To distinguish the effect of inhibiting the ILC, on appetitive versus consummatory behavior, we compared the ILC inhibition effect on the effort to get the reinforcers with that on food intake. Once the animals learned the task and they had reached a threshold of 45 lever presses to get one pellet as reward, they were subjected to a non-rewarded lever-pressing session, after a 24-h fasting or *ad libitum* feeding schedule.

**FIGURE 1:**
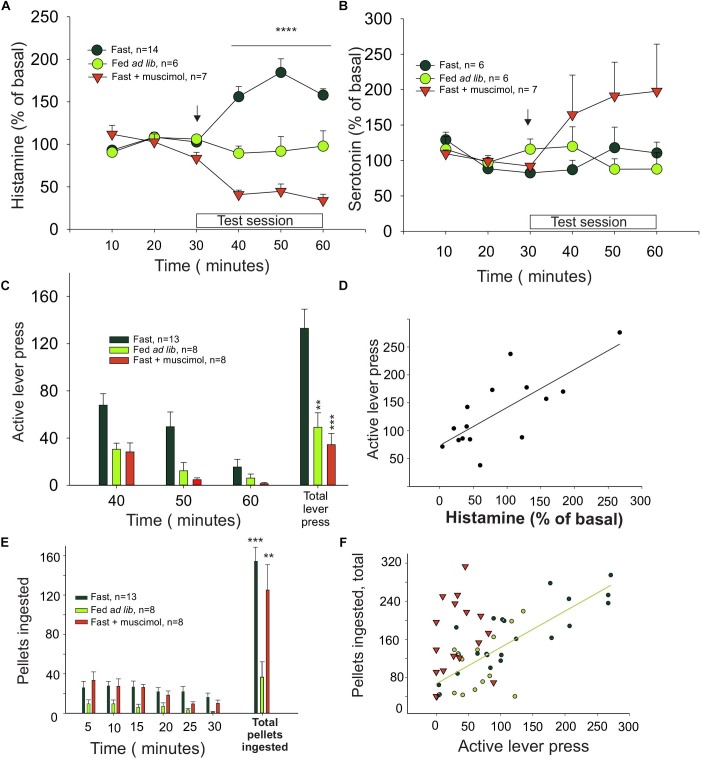
Histamine release in the medial prefrontal cortex is correlated with effort. **(A)** Percentage of extracellular histamine in relation to baseline levels (mean of 3 free samples, from 3 to 30 min) in 24-h food-restricted rats (dark green circles), in food-restricted animals with inactivated IL (inverted red triangles) after a microinjection of muscimol into the IL (signaled with an arrow), and in animals fed *ad libitum* (light green circles). The 30 min in which a light indicated food availability and animals could press a lever for food but received no food reward is indicated as test session. **(B)** Percentage of extracellular serotonin in the same groups and times shown in graph **(A)**. **(C)** Number of lever presses in food-restricted animals with intact IL (dark green bars) and in the *ad libitum* (light green bars) and muscimol (red bars) groups. **(D)** Correlation between histamine release and lever presses. **(E)** Ingestion of pellets after the test session. **(F)** Correlation between the amount of food eaten and the effort, measured as number of lever presses, that each animal made during the test session (^∗^*P* < 0.05, ^∗∗^*P* < 0.01, ^∗∗∗^*P* < 0.001, and ^∗∗∗∗^*P* < 0.0001).

Histamine levels in dialysates from the ILC of fasted rats were increased during the test session, when availability of reward was signaled (Figure 1A, black symbols, *n* = 9), compared to basal. Otherwise, in rats fed *ad libitum* (Figure 1A, green symbols, *n* = 6), histamine levels remained unchanged, indicating that motivation (hunger) determines how much histamine is released. A two-way ANOVA revealed a significant effect of time (*F*_5,90_ = 4.99, *P* = 0.0004), treatment (*F*_1,90_ = 16.56, *P* = 0.0007) and interaction time–treatment (*F*_5,90_ = 7.39, *P* < 0.0001). In contrast, serotonin levels in the same groups of rats did not change significantly during the unrewarded test session (one-way ANOVA: *P* = 0.8445), except for the muscimol-treated group, in which serotonin levels are increased after the muscimol injection in the IL (Figure 1B), in agreement with previous reports (Riveros et al., 2015).

Animals from the fasted group made three times more lever presses than the satiated rats (Figure 1C), showing that motivation also determined how much effort was displayed. Bilateral microinjection of muscimol into the ILC of hungry rats decreased the number of unrewarded lever presses compared to control, fasted animals (one-way ANOVA: *F*_2,35__=_ 12.11, *P* < 0.0001; Figure 1C) to the same level observed in the satiated rats. Therefore, inhibition of ILC decreased the effort displayed to obtain food and prevented the histamine increase observed in the fasted rats during the test (arrow in Figure 1A; red triangles, *n* = 7). Two-way ANOVA revealed a significant treatment (*F*_1,90_ = 57.64, *P* < 0.0001) and treatment–time interaction (*F*_5,90_ = 20.76, *P* < 0.0001) effect but no time effect alone (*F*_5,90_ = 0.947, *P* = 0.454).

Infusion of vehicle into the ILC of fasted rats before the test session did not induce an increase on histamine levels (two-tailed *t*-test, *P* = 0.984) or in the number of unrewarded lever presses observed in the fasted untreated rats (two-tailed *t*-test, *P* = 0.736).

We found a positive and significant correlation between the increase of extracellular histamine level and number of unrewarded lever presses in fasted rats during the test session (Pearson correlation, *r* = 0.755, *P* = 0.0011; Figure 1D), suggesting that the release of histamine may be causally related to motivation extent or the effort that a rat is willing to make to obtain food.

To evaluate if muscimol infusion in the ILC may affect food intake as a proxy of the consummatory phase of motivated behavior, we measured the amount of food intake immediately after the test session; at this point, the rats received one pellet per lever pressing. The fasted rats ate three times as much as the *ad lib* group (Figure 1E), regardless of the treatment received (muscimol, vehicle, or no treatment, one-way ANOVA; *F*_2,27_ = 12.33, *P* = 0.0002). This finding indicates that infusing muscimol into the ILC did not alter food intake, directly implying that the ILC did not influence the consummatory phase of feeding. Furthermore, we found a positive and significant correlation between the number of lever presses during the test session and the number of pellets ingested by untreated fasted rats and *ad lib* rats (black and green circles, respectively; Figure 1F) (Pearson correlation, ρ = 0.7909; *P* < 0.0001). In contrast, fasted rats treated with muscimol did not show a significant correlation (red triangles; Figure 1F, *P* = 0.4), indicating that the positive correlation between appetite and intake is lost after ILC inactivation induced by muscimol. Microinjection of muscimol into the ILC did not change the number of unrewarded lever presses during the test session in the *ad lib* rats (*t*-test vs. vehicle; *P* = 0.399; data not shown), further supporting that motivation induced by fasting determined how much effort was made by the animal to obtain food.

### Active Versus Rest Circadian Phase

To evaluate if the phase of the circadian cycle might have influenced the effort made by hungry rats to obtain food during the resting phase, the animals were tested during the active phase.

**FIGURE 2:**
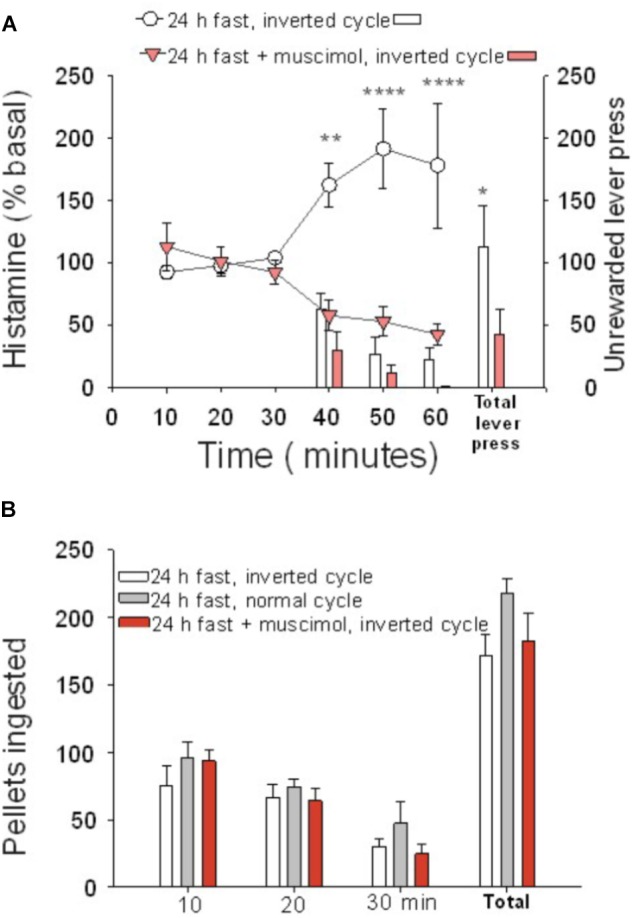
Cortical histamine levels increased during goal-directed behavior in the active phase of the day. **(A)** During the test session performed in the active phase of the day (30–60 min), the percentage of extracellular histamine in relation to baseline levels increased in 24-h food-restricted rats (white circles), but not when the IL of fasted animals was inactivated (inverted red triangles) with a microinjection of muscimol into the IL. The number of lever presses for food during the test session was higher in IL intact rats (white bars) than in IL-inactivated rats (red bars). **(B)** Number of ingested pellets was similar in three groups of 24-h food-deprived rats: IL muscimol injected and under inverted cycle (red bars), intact inverted cycle (white bars), and normal cycle (gray bars) (^∗^*P* < 0.005, ^∗∗^*P* < 0.001, ^∗∗∗^*P* < 0.0001).

Animals tested during their active phase (*n* = 8) did not show differences in behavior regarding the total number of lever presses or the temporal pattern in which the lever presses were done, compared to rats tested during the rest phase (*n* = 8). Both groups displayed more lever presses at the beginning of the test and less at the end and, irrespective of the basal levels, both groups had comparable increases in extracellular histamine levels during the 30 min of the unrewarded test (Figure 2A). The two-way ANOVA revealed a significant effect of time (*P* = 0.004) when comparing basal levels with histamine during the 30 min of the test. Animals tested during their dark phase (inverted cycle group) ingested a comparable number of pellets when tested in their light phase (normal cycle group) (Figure 2B, one-way ANOVA; *F* = 0.87548, *P* = 0.431325), regardless of having an inhibited ILC.

The basal extracellular neurotransmitter concentrations in the ILC were higher during the active than during the resting phase (*P* = 0.029 for histamine, *P* = 0.0022 for serotonin). Neurotransmitter concentrations were 0.30 ± 0.0607 and 0.16 ± 0.0206 pmol/20 μl for histamine (*n* = 31, active phase; *n* = 48, rest phase), respectively; for serotonin, the values were 0.41 ± 0.0667 and 0.17 ± 0.016 pmol/20 μl (*n* = 18, active phase; *n* = 44, rest phase), respectively. The daily variations in extracellular histamine and serotonin levels were similar to those described previously (Mochizuki et al., 1992; Barassin et al., 2002). These results indicate that it is the change in brain histamine extracellular level and not the absolute value that is relevant for appetitive behavior.

### Role of H1 Histamine Receptors on Motivation for Food

**FIGURE 3:**
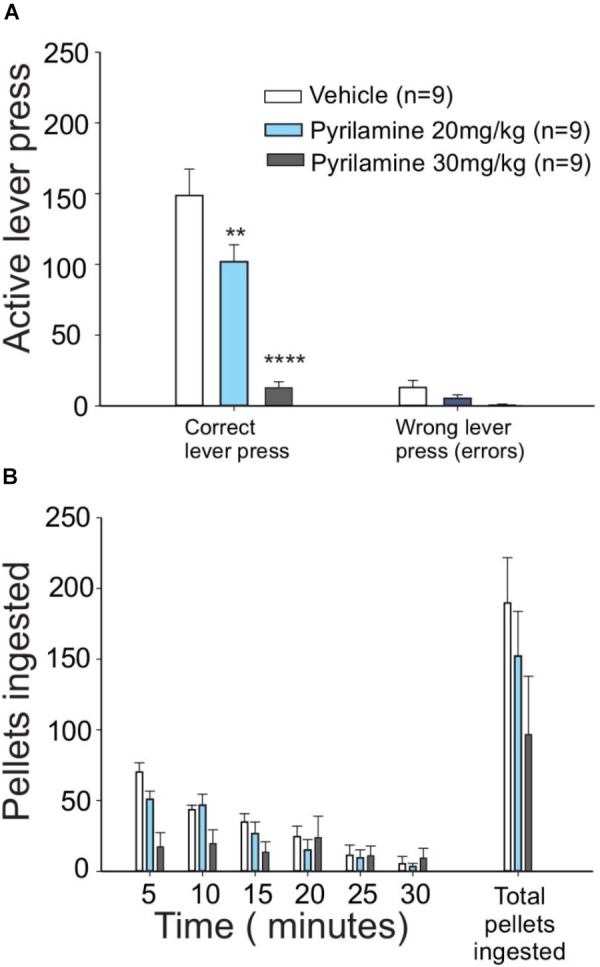
H1 antagonist pyrilamine dose-dependently reduced the effort to obtain food. **(A)** Lever presses for food were higher in vehicle (saline)-injected food-deprived rats (white bars) than in rats that received 20 mg/kg pyrilamine injected i.p. (light blue bars): pyrilamine effect was even higher in the rats given a 30 mg/kg dose (gray bars). **(B)** Pyrilamine-treated rats ate comparable amounts of pellets to the vehicle group (^∗^*P* < 0.05, ^∗∗^*P* < 0.01, ^∗∗∗^*P* < 0.001, and ^∗∗∗∗^*P* < 0.0001).

To test if the appetitive behavior induced by histamine is mediated through H1 receptors, one of the most abundant histamine receptors in the brain (Haas et al., 2008), we administered pyrilamine (an inverse agonist of H1 receptors) i.p. before the session of unrewarded lever presses. We found that pyrilamine induced a dose-dependent decrease in unrewarded lever presses (Figure 3A, one-way ANOVA; *F*_2,21_ = 19.39, *P* < 0.0001), but there was no change in the number of pellets ingested immediately after the test (Figure 3B). These results suggest that the H1 histamine receptor is involved in the effort made by fasted rats to obtain food. The number of errors (an error consisted of pressing the no-reward lever) in the test did not differ between the treatments (one-way ANOVA; *F* = 0.4033, *P* = 0.088); this finding points to a mild or absent sedative effect in pyrilamine-treated rats at the dose we used.

## Discussion

In this study, we provide novel evidence of the pivotal role of histaminergic signaling through H1 in the degree of effort displayed during the appetitive phase of motivated behavior. Additionally, we showed that the ILC is necessary to promote an increase in the histamine release during the appetitive phase of motivated behavior. We propose that ILC-TMN conforms to a circuit that is shown to be fundamental in regulating effort investment during goal-directed behaviors.

We evaluated motivation using instrumental responses since these responses are considered a direct assessment of motivation in animals to obtain a reward or avoid punishment (Berridge and Robinson, 1998; Baldo and Kelley, 2007). As expected, hungry rats displayed more effort to obtain food rewards than the *ad lib* rats while they also had an increased histamine release during the instrumental conditioning test session. Hungry rats with bilateral ILC inactivation showed a decrease in histamine level and reduced effort to get food rewards compared with the untreated hungry rats. In fact, hungry rats with inactive ILC showed the same diminished motivation for food and histamine levels as the *ad lib* group.

Nevertheless, when fasted rats were allowed to acquire a pellet immediately after the test session easily, they behave accordingly to their internal state: the two fasted groups ate nearly three times more rewards than the *ad lib* rats. Thus, instrumental responses were sufficient to distinguish between the effects of these manipulations on appetitive versus consummatory stages of the behavior. ILC inhibition dissociated food intake from the motivation to obtain food as shown in Figure 1F.

The synchronous increase in the extracellular histamine level and motivation observed in the fasted rats was similar during the resting and active phases, indicating that histamine is not merely a wakefulness signal but rather that its increase is essential for motivation during the day and night.

The results shown here do not focus on pinpointing the specific brain locations where the activation of H1 receptors mediates the increase in behavioral arousal, locomotor activity, or sympathetic activation. However, activation of these receptors in the cortex and thalamus as well as in hypothalamic and brainstem nuclei could mediate behavioral arousal (Panula and Nuutinen, 2013). Similarly, histamine acting on motor cortices and striatum could mediate the increase in locomotor activity (Inoue et al., 1996). Histamine could also, directly and indirectly, activate sympathetic nuclei (Valdés et al., 2010; Murakami et al., 2015) and also act on the rest of the AAS promoting the release of other neuromodulators such as dopamine, noradrenaline, or acetylcholine as it has been proposed before (Valdés et al., 2005; Torrealba et al., 2012).

Changes in expected reward can induce frustration that stems from an unexpected omission of an expected reward or a reduction in the reward magnitude (Amsel, 1994). In turn, the emotional state of frustration may possibly impact on instrumental responses (Amsel, 1994). Then, it is noteworthy that the test we used to assess motivation (lever pressing for food without reward), as the animal expect a reward that it is not getting, may induce frustration. Therefore, the histamine release we observed during the test may be frustration-related since the rats obtained no reinforcers despite their efforts. However, we found that histamine release was positively correlated with the instrumental response (Figure 1D). If the observed histamine release were indeed related to frustration, we hypothesize that a negative correlation would be more likely, considering the negative emotional impact that a reduction in the expected reward would have in the output of the instrumental behavior observations (Scull et al., 1970; Flaherty et al., 1983, 1995). These considerations rather suggest that histamine release is not secondary to frustration. Similarly, we previously reported that the increase in histamine release during unreachable food presentation is not likely to be part of a stress response, because this manipulation is not associated to signs of stress in the brain (Valdés et al., 2010), which is in concordance with the notion that in natural conditions, an animal has to work to obtain food. Since we have not used H2 or H3 receptors blockers, we do not have information about participation of these receptors in the histamine-mediated components of food-directed behavior, and it would be interesting to evaluate in a future research how each of the receptors is collaborating in the increase of arousal mediated by histamine.

Motivation, the invigoration of behavior, and the reinforcement of adaptive behaviors, such as instrumental goal-seeking behavior, have been extensively associated with the mesocorticolimbic systems and dopamine (Robbins et al., 1989; Berridge and Robinson, 1998). In fact, the relevance of arousal to effort-related functions has been extensively addressed by Salamone et al. (2007). A simple way to link the motivational functions of the mesocorticolimbic systems with our hypothesis that the TMN and histamine are also crucial elements in motivation is to consider the IL-TMN circuit as an integral part of the motive circuit with the prefrontal cortex-basal ganglia-thalamus loops involved in goal-directed behavior (Alexander et al., 1986; Groenewegen et al., 1990; Pierce and Kalivas, 1997; Zahm, 2000; Koob, 2003). Within this framework, the IL-TMN circuit explicitly contributes to motivation by increasing arousal and sympathetic activity (Valdés et al., 2006) to prepare the brain and body for appetitive behavior. However, the anatomical and functional relationship between the histaminergic system and the dopaminergic input to the nucleus accumbens to induce arousal and to modulate effort-based decisions (Salamone et al., 2007) remains to be explored. Also, there are interactions between histamine and dopamine at the neurochemical level that deserve further investigation (Brabant et al., 2010).

We measured serotonin levels on the same samples in which histamine was measured. We observed no changes in serotonin release while fasted animals were pressing the lever to obtain food reward, suggesting that serotonin signaling is not mediating the incentive motivation. Furthermore, serotonin was increased in the animals that were injected with muscimol in the ILC. Remarkably, lever pressing or effort to obtain food reward is reduced in these animals, in keeping with the hypothesis of the role of serotonin in the reduction of motivation for food (Hutson et al., 1986). This reduction in motivation, observed as a reduction in operant responding for food, is mediated by serotonin release in the ventral tegmental area, suggesting that serotonin action opposes to dopamine mediation of motivation at this site (Browne et al., 2019). In this regard, our work suggests that histaminergic and serotoninergic systems regulate dopaminergic mediation of incentive motivation in an opposite way.

### Clinical Implications

IL activation could be promoting the activation of histaminergic TMN neurons leading to histamine release and increased arousal and disposition to work, allowing the optimal unfolding of appetitive behavior. In some conditions such as apathy or fatigue, this optimal unfolding is disrupted because of increased effort perception (Pageaux et al., 2015).

Apathy can be defined as a reduction of self-generated, voluntary, and purposeful behaviors (Marin, 1996; Robert et al., 2010; Chase, 2011). Dysfunction of the IL-TMN axis may underlie the auto-activation type of apathy observed in individuals with lesions to frontal cortical areas including Brodmann area 25/32 (Levy and Dubois, 2006), the primate homologous to the rat IL (Ongür and Price, 2000).

On the same line, the mental fatigue that people may experience after or during prolonged periods of cognitive activity (Boksem and Tops, 2008) could be related to an IL-TMN circuit alteration. Aversion to a further investment of effort in task performance is central to mental fatigue. For instance, mental fatigue limits exercise tolerance in humans through the higher perception of effort rather than cardiorespiratory and muscular mechanisms (Marcora et al., 2009). It is not unlikely that the IL-TMN axis dysfunction plays a role in mental fatigue, possibly by increasing effort perception.

We suggest that devising methods to advantageously modulate IL-TMN axis activity may contribute to the treatment of different conditions that have reduced motivation as a central symptom.

## Conclusion

The amount of effort the rats invest to obtain palatable food reinforcers depends on the activity of the ILC and the release of histamine acting on H1 receptors.

## Ethics Statement

This study was carried out in accordance with the recommendations of the Guide for the Care and Use of Laboratory Animals; National Research Council, Comité Ético Científico para el Cuidado de Animales y Ambiente. The protocol was approved by the Comité Ético Científico para el Cuidado de Animales y Ambiente.

## Author Contributions

All authors listed had contributed substantially to the work. MR and FT designed the experiments and wrote the first draft. MR conducted the experiments. MF and JV collaborated in data processing and editing of the mansuscript.

## Conflict of Interest Statement

The authors declare that the research was conducted in the absence of any commercial or financial relationships that could be construed as a potential conflict of interest.
